# Application of Omics Technologies for Evaluation of Antibacterial Mechanisms of Action of Plant-Derived Products

**DOI:** 10.3389/fmicb.2016.01466

**Published:** 2016-09-27

**Authors:** Bruno S. dos Santos, Luís C. N. da Silva, Túlio D. da Silva, João F. S. Rodrigues, Marcos A. G. Grisotto, Maria T. dos Santos Correia, Thiago H. Napoleão, Márcia V. da Silva, Patrícia M. G. Paiva

**Affiliations:** ^1^Departamento de Bioquímica, Centro de Biociências, Universidade Federal de PernambucoPernambuco, Brazil; ^2^Programa de Pós-graduação em Biologia Parasitária, Universidade CEUMAMaranhão, Brazil; ^3^Centro de Tecnologias Estratégicas do NordestePernambuco, Brazil; ^4^Instituto Florence de Ensino SuperiorMaranhão, Brazil

**Keywords:** natural products, antimicrobial target, genomics, transcriptomics, proteomics, metabolomics

## Abstract

In the face of increasing bacterial resistance to antibiotics currently in use, the search for new antimicrobial agents has received a boost in recent years, with natural products playing an important role in this field. In fact, several methods have been proposed to investigate the antibacterial activities of natural products. However, given that the ultimate aim is future therapeutic use as novel drugs, it is extremely necessary to elucidate their modes of action, stating the molecular effects in detail, and identifying their targets in the bacterial cell. This review analyzes the application of “omics technologies” to understand the antibacterial mechanisms of bioactive natural products, to stimulate research interest in this area and promote scientific collaborations. Some studies have been specifically highlighted herein by examining their procedures and results (targeted proteins and metabolic pathways). These approaches have the potential to provide new insights into our comprehension of antimicrobial resistance/susceptibility, creating new perspectives for the struggle against bacteria, and leading to the development of novel products in the future.

## Introduction

The alarming spread of bacterial resistance to antibiotics is one of the most serious challenges to global public health, as drug resistance has been found for all classes of antibiotics used in clinical practice (Arias and Murray, 2015). Moreover, few new antibiotic classes have been discovered in the last several decades. Managing this situation will require extensive search for new drugs and elucidation of their mechanisms of action (MOA; Kon and Rai, 2012).

In general, natural plant products have been documented as important alternative sources of new antimicrobial agents. Traditional knowledge regarding the use of medicinal plants has driven the identification of plant-derived products with different chemical structures (Radulovic et al., 2013). This structural diversity enables the active phytochemicals to act through diverse mechanisms (usually targeting multiple biochemical pathways), which are sometimes different from those used by traditional antibiotics (Simões et al., 2009; Radulovic et al., 2013; Harvey et al., 2015).

Given the great achievements in the development of analytical tools for identification and isolation of antimicrobial phytochemicals, the biggest challenge now is the characterization of their MOA, which is essential to supporting their use as lead molecules for drug-development programs (Swinney and Anthony, 2011). The approaches adopted to identify a possible drug target are predominantly based on the combined application of several biochemical and genetic assays (such as flow cytometry, electron microscopy, colorimetric assays, and gene expression analysis), using both cellular systems and model organisms. However, the combination of these techniques takes time and requires the use of large amounts of the compound under investigation (Swinney and Anthony, 2011; Schirle et al., 2012; Sianglum et al., 2012; Roemer and Boone, 2013; Tang, 2015).

**Figure 1:**
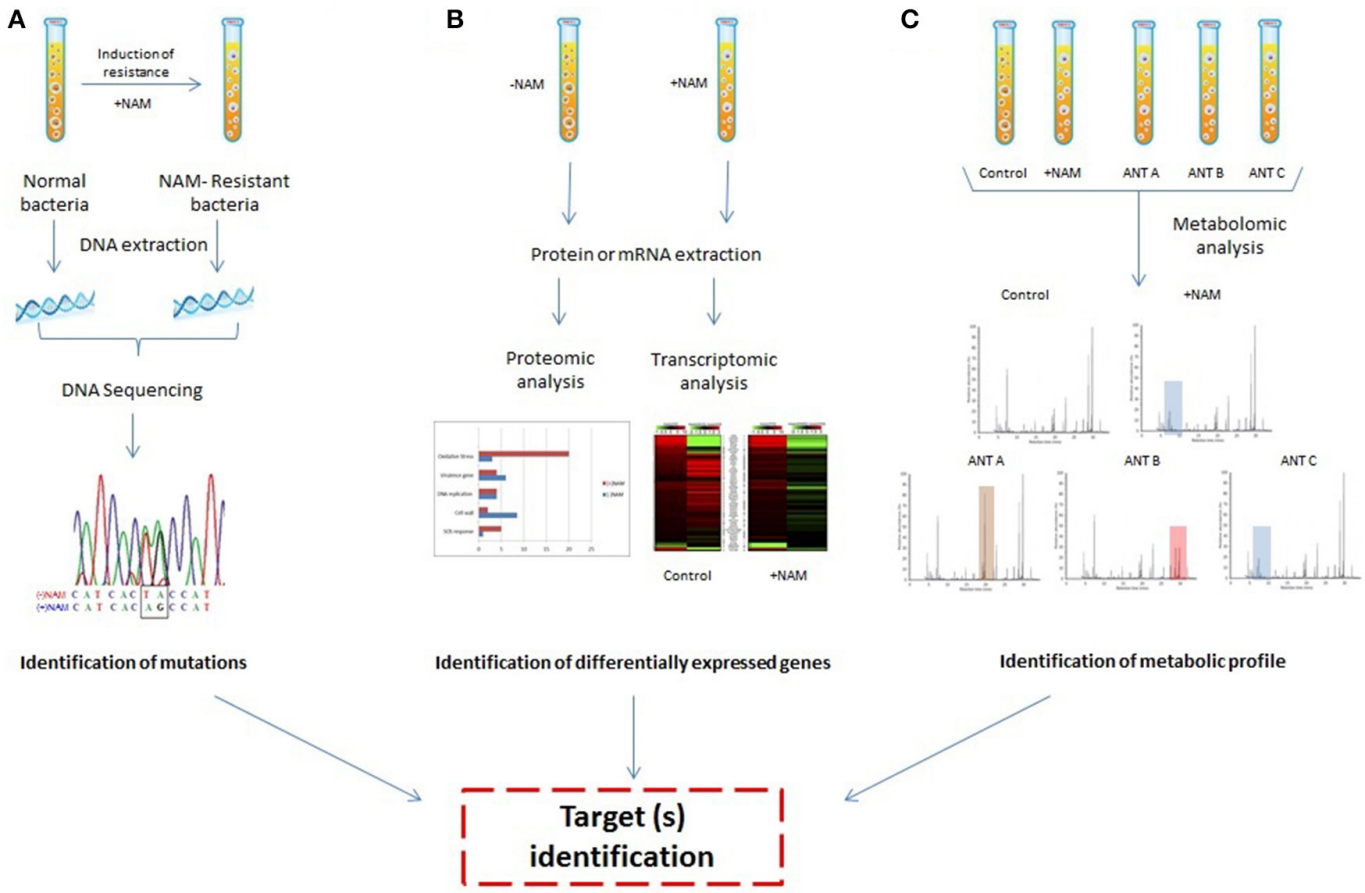
**Overview of applications of “omics technologies” to evaluate the mechanisms of action of natural antimicrobials (NAM)**. **(A)** Genomic approaches: the target can be discovered by comparing the DNA sequence of a NAM-resistant bacteria and normal bacteria. **(B)** Proteomic and transcriptomic approaches: target identification can be performed by evaluating differential expression of genes in strains treated or untreated with NAM. **(C)** Metabolomic analysis: the metabolic profile of a bacterium treated with NAM can be compared with the profile of different drugs with known actions, potentially leading to target identification.

Recent advances in “omics” technologies (genomics, transcriptomics, proteomics, and metabolomics) are attributed to innovative breakthroughs in genome sequencing, bioinformatics, and analytic tools such as liquid and gas chromatography and mass spectrometry, along with high-throughput technologies. Omics technologies have provided crucial insights into processes related to bacterial physiology, virulence, stress, and the MOA of antimicrobial compounds (Roemer and Boone, 2013; Tang, 2015; Figure 1). The use of these tools provides deeper and more robust data, and has greater potential to reveal new therapeutic targets than conventional assays. These new targets and their related pathways are critically important in the struggle to overcome drug resistance (Roemer and Boone, 2013). In this review, we will focus on the application of these methodologies to study the mechanisms of action of plant-derived antibacterial compounds.

## Overview of phenotypical methods for the elucidation of antibacterial MOA

Compounds with antibacterial activity are usually selected by traditional antimicrobial methodologies such as agar-diffusion based assays, determination of minimum inhibitory (MIC), and bactericide (MBC) concentrations, and time-kill curves. Once an antimicrobial candidate is selected, the next step is to characterize its action on bacterial structures such as the cell membrane, genetic material, and/or protein synthesis, usually through phenotypical assays (Gottschalk et al., 2013; Silva et al., 2013a; Monte et al., 2014; Hyldgaard et al., 2015; Magi et al., 2015; Gerits et al., 2016; Moreira et al., 2016). Next, the molecular pathway can be elucidated by analyzing the expression of the genes related to the phenotype observed (Gottschalk et al., 2013). Here, we will discuss the methods to evaluate the effects on the cell membrane, DNA damage, and macromolecular synthesis.

The morphological alterations on cell membrane can be efficiently studied by electron microscopy (Silva et al., 2013a). Alternatively, various assays are performed to evaluate membrane integrity. One simple assay measures the leakage of cell material that absorbs at 260 nm wavelength on the supernatant of treated cells. Nucleic acids and related compounds (such as pyrimidines and purines) have max UV light absorbance at this wavelength. The increase of this material in the supernatant indicates bacterial cell wall and/or membrane damage (Chusri and Voravuthikunchai, 2009; Silva et al., 2013b). Another useful approach is the detection of cytoplasmic enzymes or ATP leakage in the cell culture supernatant of treated bacteria (Gottschalk et al., 2013). Fluorescent probes are also widely used to evaluate membrane viability, such as propidium iodide (PI), SYTOX green, 1-*N*-phenylnaphthylamine (NPN), and 3,3′-diethyloxacarbocyanine iodide [DiOC_2_(3)]. For example, PI is used to evaluate membrane integrity, as it is a membrane impermeant dye that intercalates DNA (only if there is a damage; Van Nevel et al., 2013); whereas DiOC_2_(3) helps measure the membrane potential (Moreira et al., 2016).

Recently, the effects of boromycin (a polyether macrolide antibiotic isolated from *Streptomyces antibioticus*) on mycobacterial cell membrane were evaluated. The authors adopted an approach where, to assess the release of cell content, a strain of *Mycobacterium bovis* was transformed with a plasmid (pGMEH-P38-mRFP) encoding mCherry Red Fluorescent Protein (mRFP). After treatment with boromycin, the fluorescence in the supernatant was measured (Moreira et al., 2016).

The action of a compound on DNA integrity can be evaluated by DNA-binding analysis, in which a sample of purified DNA (usual plasmid DNA) is mixed with different concentrations of the tested compound. After an incubation period, the reaction is subjected to electrophoretic analysis. From the gel retardation assay, interference on the migration profile in relation to control cells is considered a positive result (the compound can bind to the DNA; Gottschalk et al., 2013). DNA fragmentation induced by an antimicrobial can be detected *in situ* by TUNEL (terminal deoxynucleotidyl transferase mediated dUTP nick end labeling) assay. Usually this assay employs fluorescently tagged dUTP, which binds to 3′-OH groups in DNA breaks (Rohwer and Azam, 2000). Difference in DNA distribution between treated and untreated cells can be analyzed using DAPI dye (4′,6-diamidino-2-phenylindole; Kjelstrup et al., 2013). Damage of bacterial DNA induces formation of the RecA filament, leading to auto-cleavage of LexA resulting in activation of SOS response. In this sense, perturbations in DNA integrity can be indirectly assessed through the expression of *recA* gene (Gottschalk et al., 2013; Kjelstrup et al., 2013).

To investigate the effects of a compound on bacterial macromolecular synthesis, a scintillation assay can be performed. In this assay, bacterial culture is labeled by the addition of radioactive precursors of DNA ([methyl-^3^H]thymidine), RNA ([5,6-^3^H]uridine), or protein (l-[G-^3^H]glutamine) synthesis. After the experimental treatment, the radiolabeled incorporation is measured using a scintillation counter (Gottschalk et al., 2013; Kjelstrup et al., 2013; Gerits et al., 2016).

## Genomics and advances in antibiotics research

The advent of efficient genetic sequencing technologies enabled the complete decoding of various microbial genomes, making all this information accessible. This resulted in the identification of a range of genes related to essential processes for bacterial survival, virulence, and mutagenesis. These genes and their products are therefore potential new antimicrobial targets (Roemer and Boone, 2013; Scheffler et al., 2013). This knowledge is of utmost importance to the development of new screening platforms for identification and selection of compounds targeting specific essential genes based on protein interactions and mutant libraries (Zhang et al., 2015; Morita et al., 2016). A classical approach, applicable to select compounds that are able to interact with a specific target, is based on affinity chromatography systems. In these assays, the target can be immobilized on a matrix (affinity matrix), and a bioactive compound that is able to bind the target can be isolated and identified (Sakamoto et al., 2012).

The interaction of a target and bioactive compound can also be evaluated using bacterial two-hybrid (BTH) systems. Several BTH platforms have been engineered, such as the BTH assay based on adenylate cyclase *(cya)* reconstitution. This system was comprehensively revised by Battesti and Bouveret (2012). Briefly, the adenylate cyclase from *Bordetella pertussis* is divided in two sub-domains (T18 and T25), and each gene encoding the proteins of interest is cloned into a vector fused to either T18 or T25 protein fragments. The vector is inserted in an *Escherichia coli* strain lacking endogenous *cya* (*cya*−). The interaction between the proteins of interest brings into proximity the T18 and T25 fragments and a Cya+ phenotype is created. This results in cyclic adenosine monophosphate (cAMP) production and consequently, in the activation of cAMP-regulated promoters (e.g., the *lac* promoter). A BTH based on *cya* activity was employed by Kjelstrup et al. (2013) to select compounds capable of disrupting the interaction between proteins involved in DNA replication of *Staphylococcus aureus*. The authors found two peptide inhibitors of the dimerization of the β-sliding clamp of the replisome.

Another system developed from genomic technologies is based on the screening of a genome-wide mutant library (Gray et al., 2015). These mutants can be generated through mutagenesis mediated by mobile genetic elements such as transposons (Pasquina et al., 2015; Gerits et al., 2016). Next, mutants with altered sensitivity to the compound are identified, elucidating the pathways involved in its action (through genomic studies). This approach was adopted to elucidate the molecular mechanism of alfalfa snakin-1 (MsSN1), an antimicrobial peptide produced by *Medicago sativa*, against *Pseudomonas fluorescens* Pf-5. This work revealed that MsSN1 peptide acts on adhesion properties of *P. fluorescens* (Ayub et al., 2015; Table 1).

**Table 1:** **Plant-derived products with mechanisms of action evaluated by “omics technologies”**.

**Phytochemical class**	**Chemical structure**	**Antimicrobial agent**	**Pathogen**	**Methodology**	**Main target(s)**	**References**
Phenolic acid	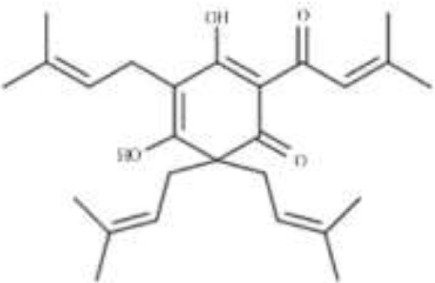	Lupulone from *Humulus lupulus*	*Mycobacterium tuberculosis*	Microarray	Surface-exposed lipids, cytochrome P450 enzymes, PE/PPE multigene families, ABC transporters, and protein synthesis	Wei et al., 2014
Chalcone	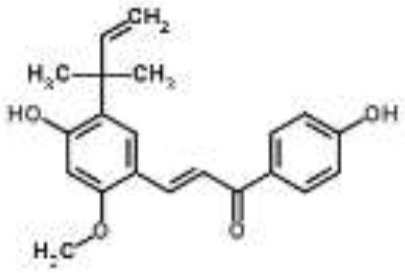	Licochalcone A from *Glycyrrhiza inflata*	*S. aureus*	Microarray	Autolysis-associated proteins, cell wall proteins, pathogenic factors, protein synthesis, and capsule synthesis	Shen et al., 2015
Terpenes	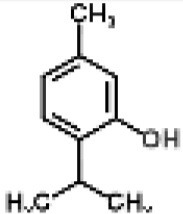	Thymol from *Thymus vulgaris*	*Salmonella enterica* serovar Thompson	2-DE analysis	Bacterial envelope and citrate metabolic pathway	Di Pasqua et al., 2010
	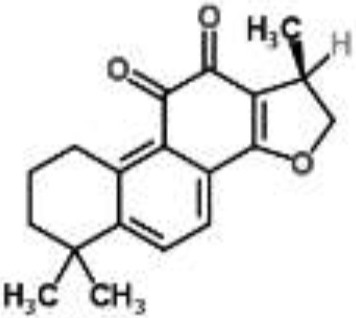	Cryptotanshinone from *Salvia miltiorrhiza*	*S. aureus*	Microarray	Oxygen radical generation	Feng et al., 2009
	–	Essential oils from *Citrus sinensis*	*S. aureus*	Microarray	Cell envelope	Muthaiyan et al., 2012
	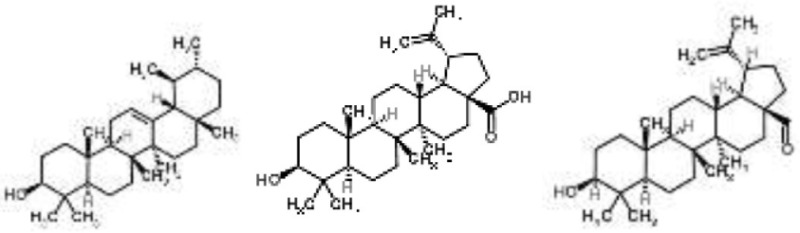	Pentacyclic triterpenoids (α-amyrin, betulinic acid, and betulinaldehyde) from *Callicarpa tomentosa*	*S. aureus*	Microarray	Bacterial cell membrane, cessation of protein synthesis, and fatty acids	Chung et al., 2013
	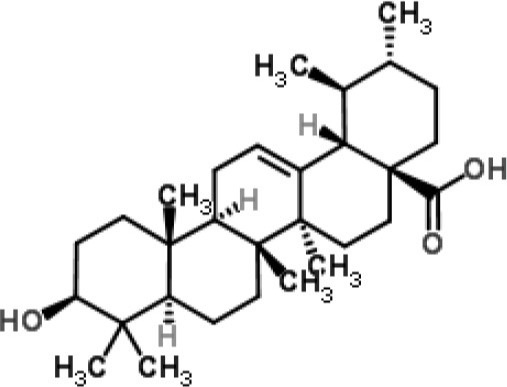	Ursolic acid	*Staphylococcus aureus*	RNA-Seq-based	Inhibition of metabolism of some amino acids and the expression of adhesins	(Qin et al., 2014)
Lignan	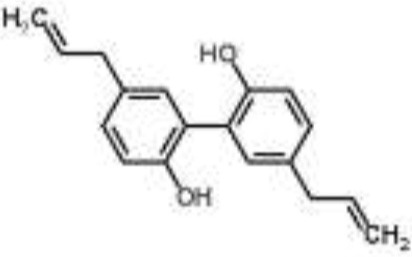	Magnolol from *Magnolia obovata*	*S. aureus*	Microarray	Virulence pathways	Wang et al., 2011
Alkaloids	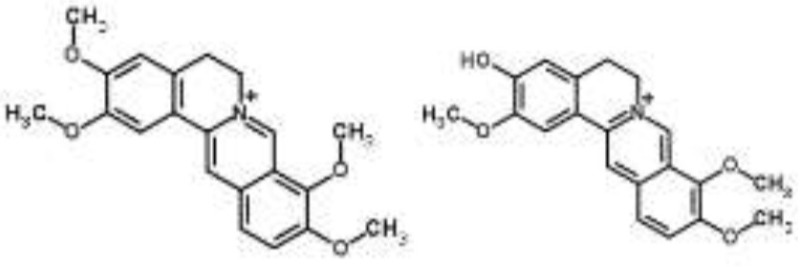	Palmatine and jatrorrhizine from *Tinospora capillipes*	*S. aureus*	HPLC-ESI/MS	RNA polymerase, gyrase, and topoisomerase IV (similar to rifampicin and norfloxacin)	Yu et al., 2007b
	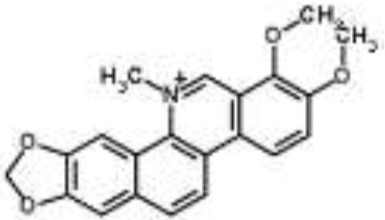	Chelerythrine from *Chelidonium majus*	*Mycobacterium tuberculosis*	Microarray	Urease, surface exposed lipids, the heat shock response, and protein synthesis	Liang et al., 2011
	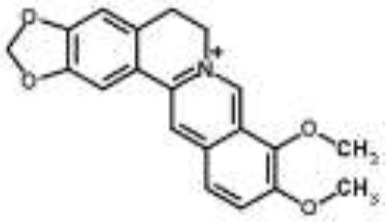	Berberine-containing plant extract from *Papaver polychaetum*	*Escherichia coli*	2-DE analysis	Elongation factor-Ts, ABC transporter, energy metabolism	Ozbalci et al., 2010
	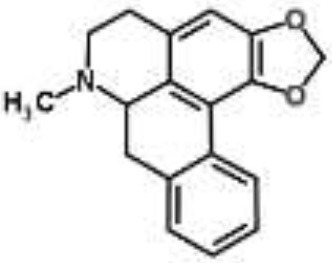	Roemerine from *Papaver rhoeas*	*Escherichia coli*	2-DE analysis	Inhibition of membrane permeability and sugar transporter proteins involved in carbohydrate metabolism	Gokgoz and Akbulut, 2015
	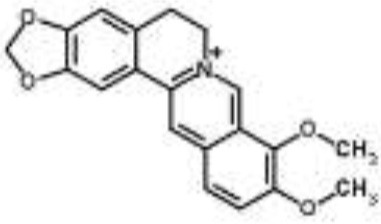	Berberine from *Aquilegia oxysepala*	*S. aureus*	HPLC/ESI-MS with PCA	Nucleic acids: same profile as rifampicin and norfloxacin	Yu et al., 2007a
	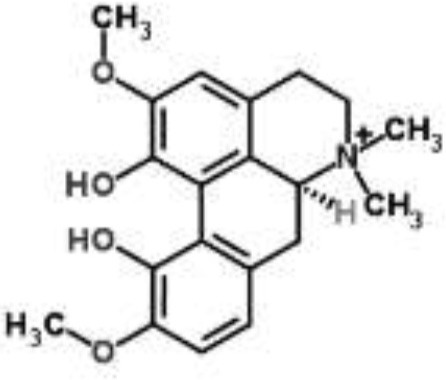	Magnoflorine from *Aquilegia oxysepala*	*S. aureus*	HPLC/DAD/ESI-MS analysis combined with PCA	Protein synthesis	Yu et al., 2007a
	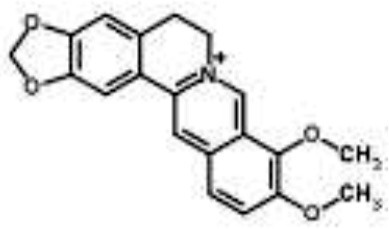	Berberine from *Coptis chinensis*	*S. aureus*	Microarray	Transport proteins	Wang et al., 2008
			*Yersinia pestis*	Microarray	Regulation of central metabolism and disruption of membrane	Zhang et al., 2009
		Berberine chloride	*E. coli*	Microarray and 2-DE	Cell division, motility, and transport processes	Karaosmanoglu et al., 2014
			*Shigella flexneri*	Microarray	DNA replication, cell division, and chromosome partitioning, bacterial cell membrane	Fu et al., 2010
Crude extract rich in esters, ketone and phenol derivative	–	Flower extract from *Melastoma candidum*	*Escherichia coli* and *S. aureus*	Denaturing gel electrophoresis and MALDI TOF-TOF MS	Glutamate decarboxylase, elongation factor-Tu, and α-hemolysin	Wong et al., 2014
Acylphloroglucinol	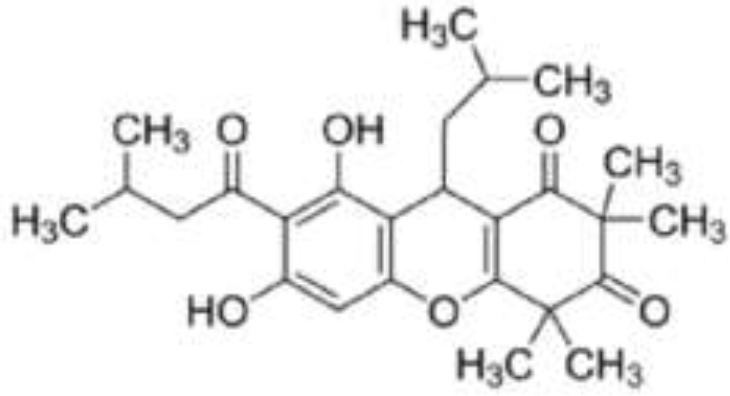	Rhodomyrtone from *Rhodomyrtus tomentosa*	*S. aureus*	2-DE analysis	Cell wall biosynthesis, cell division, protein degradation, Stress response, virulence factors, energy production, and macromolecule biosynthesis	Sianglum et al., 2011
				Microarray	Biosynthesis of amino acids, cell envelope, protein transporters, and nucleotide metabolism	Sianglum et al., 2012
			*S. pyogenes*	2-DE analysis	Virulence genes, glyceraldehyde-3-phosphate dehydrogenase, cAMP factor, streptococcal pyrogenic exotoxin C	Limsuwan et al., 2011
Anthraquinone	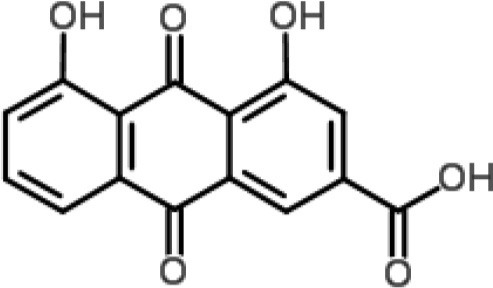	Rhein from *Rheum palmatum*	*S. aureus*	Microarray	Cellular transport	Yu et al., 2008
Naphthoquinone	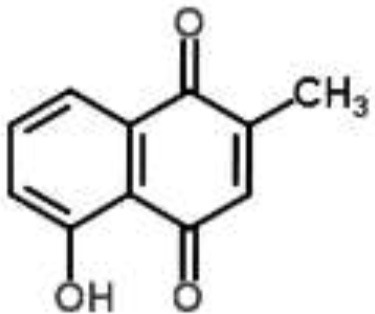	Plumbagin from *Plumbago zeylanica*	*B. subtilis*	2-DE and iTRAQ	Citric acid cycle and heme biosynthesis	Reddy et al., 2015
	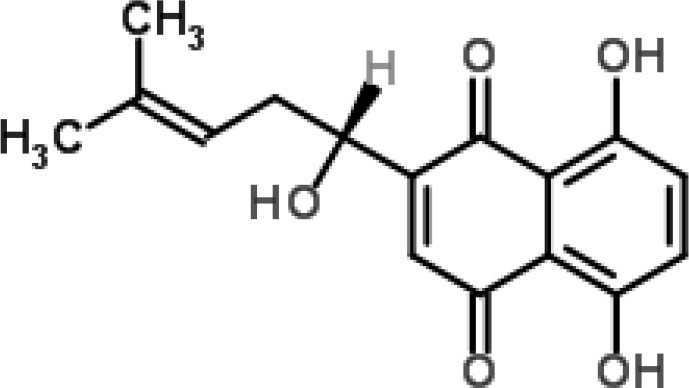	Shikonin-containing plant extract from *Lithospermi radix*	*S. aureus*	2-DE	Helicase (RuvB) and bacterial cell wall biosynthesis	Lin et al., 2012
Phytoanticipin	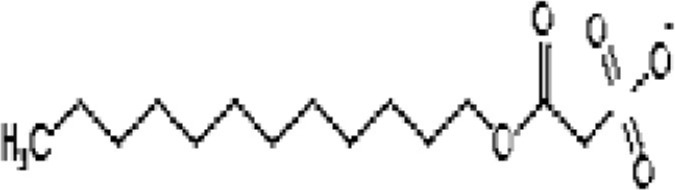	Sodium Houttuyfonate from *Houttuynia cordata*	*S. aureus*	Microarray	Autolysin pathway	Liu et al., 2011
Catechin	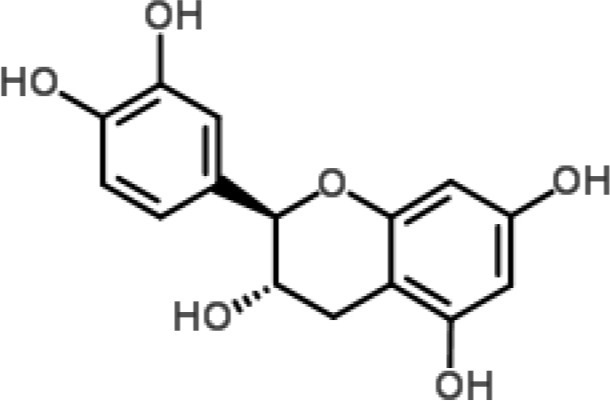	Tea polyphenols (especially catechins) from *Camellia sinensis*	*P. aeruginosa* and *S. marcescens*	2-DE and MALDI-TOF/TOF analyzes	Membrane metabolism	Yi et al., 2010, 2014
Stilbene	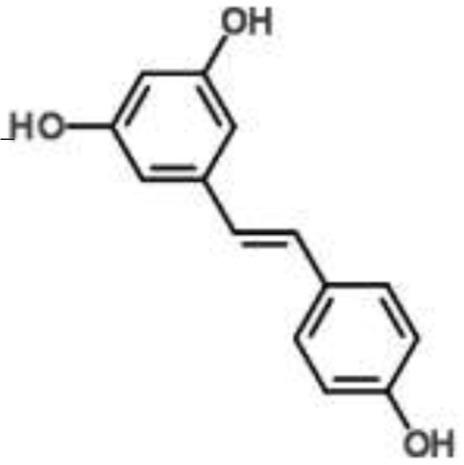	Resveratrol	*Staphylococcus aureus*	RNA-Seq-based	Inhibition of quorum sensing, synthesis of surface protein, and capsular polysaccharides	Qin et al., 2014
Peptide	–	Alfalfa snakin-1 (MsSN1; several plants)	*Pseudomonas fluorescens*	Screening of genome-wide mutant library	Adhesion properties	Ayub et al., 2015

Sequencing methods can also be applied in the discovery of potential targets through the selection of mutants resistant to the new compound, followed by genetic comparison (Brazas and Hancock, 2005; Bachmann et al., 2014; Köser et al., 2014). In this case, the wild type strain is cultivated in the presence of the antimicrobial compound, and mutants can be generated by natural selection. This strategy can be exemplified by the elucidation of the antibacterial action mechanism of a new compound designated SPI031 [*N*-alkylated 3, 6-dihalogenocarbazol 1-(sec-butylamino)-3-(3,6-dichloro-9H-carbazol-9-yl)propan-2-ol] against *P. aeruginosa*. The authors selected three SPI031-resistant mutants and compared their whole genome sequences with the genome of *P. aeruginosa* wild-type (WT). They found mutations in the genes coding for the multidrug efflux pump (*nfxB*) and outer membrane synthesis (*htrB* and PA14_23400; Gerits et al., 2016). This approach has the advantage of being able to overcome the limitations of protein interaction assays or mutant libraries in revealing new targets (Nijman, 2015).

## Transcriptomics technologies in antibiotic research

Several studies support that the MOA of an antibiotic is related to the interaction of multiple pathways (Dwyer et al., 2015). Thus, techniques for the analysis of gene expression on a large scale (transcriptional analysis) have been widely applied to evaluate the action mechanisms of an antibiotic, including those derived from plants. These assays are classified as “transcriptomics,” which is a set of high-throughput technologies that analyze the transcriptome (the complete set of RNA transcripts produced by an organism or cell under specific circumstances; Nambiar et al., 2010).

Currently, DNA microarrays are the technology of choice for large-scale studies of gene expression. Microarray technology was developed using the information available from the genome projects and is based on the hybridization of cDNA (complementary DNA produced from mRNA) to oligonucleotide probes incorporated into a slide. Each probe has a sequence of a specific gene from the organism. Microarrays have the advantage of simultaneously measuring the global gene expression at the level of transcription, and thus providing insights into the molecular pathways related with the investigated phenomena. Generally, at least two different experimental situations are studied and the cDNA extracted from each is labeled with different fluorophores (Smith et al., 2005; Nagaraj and Singh, 2010). Microarrays have been used to elucidate the MOA of several phytochemicals, opening new insights into the modulation of different pathways caused by an antimicrobial agent (Hammami and Fliss, 2010; Chung et al., 2013; Wei et al., 2014; Eng and Nathan, 2015; Shen et al., 2015). We highlight some of them herein, with more examples provided in Table 1.

Licochalcone A is a chalcone isolated from *Glycyrrhiza inflata* (Fabaceae), which shows different pharmacological activities, including the inhibition of *S. aureus* biofilm formation and planktonic cells. The transcriptional profile of this pathogen treated with licochalcone A was evaluated by a microarray assay. Several genes had their expression significantly altered after licochalcone A treatment, especially those related to autolysis, cell wall, pathogenic factors, protein synthesis, and capsule synthesis (Shen et al., 2015).

Another example is a study carried out to evaluate the molecular effects triggered by lupulone (phenolic acid from *Humulus lupulus*) in *Mycobacterium tuberculosis*. The authors reported that 540 genes were found to be differentially regulated, which were involved in various pathways such as surface-exposed lipids, cytochrome P450 enzymes, PE/PPE multigene families, ABC transporters, and protein synthesis (Wei et al., 2014).

Pentacyclic triterpenoids (such as α-amyrin, betulinic acid, and betulinaldehyde) are phytochemicals with recognized anti-*S. aureus* action. The transcriptional changes induced by sub-inhibitory doses of these three components in a methicillin-resistant *S. aureus* strain were analyzed by microarray, showing that they act by targeting pathways such as cell division (FtsZ), ABC transporters (OPP-1C), fatty acid (fabz), and peptidoglycan (fmhB, PBP2) biosynthesis, DNA replication (ccrA), two-component regulatory system, and β-lactam resistance (mecR1). The proposed MOA was the inhibition of cell growth by destabilization of the bacterial cell membrane combined with the cessation of protein and fatty acids synthesis (Chung et al., 2013).

Transcriptional analysis by microarrays and other gene probe-based methods are relatively inexpensive and compatible with high-throughput studies. However, these methods have some limitations, such as the availability of arrays for a particular species (the target species needs to have a sequenced genome), and technical issues including reproducibility. Specifically for bacterial studies (or for other highly variable genomes), another discovered issue is that the array is designed using genomic information of a wild type strain and cannot detect genes present only in a particular isolate (Wang et al., 2009; Bumgarner, 2013; Dopazo, 2014; Zhao et al., 2014). These factors together make sequence-based approaches an interesting alternative for measuring gene expression. These methods quantify gene expression directly through sequencing the cDNA produced from a fragment that maps transcript. The most recent sequence-based method, RNA sequencing (RNA-Seq), employs recently developed technologies, providing a deeper quantification of gene expression (Rapaport et al., 2013).

RNA-Seq studies have been performed to evaluate the MOA of some plant-derived products (Table 1). For example, this method was employed to analyze the molecular pathways involved in the action of ursolic acid and resveratrol against the biofilm produced by methicillin-resistant *S. aureus* (Qin et al., 2014). Ursolic acid is a pentacyclic triterpenoid found in numerous classes of medicinal plants (Kashyap et al., 2016). The anti-biofilm action of ursolic acid was found to be related to a reduction in the expression of genes involved in amino acid metabolism and adhesin expression (Qin et al., 2014). Conversely, resveratrol (a polyphenol found in red wine) was found to act through the inhibition of pathways related to quorum sensing, surface proteins, and capsular polysaccharides (Qin et al., 2014).

## Proteomics assays

Proteomics is the systematic evaluation of all proteins expressed by one particular cell, tissue, biological fluid, or organism in a given time period. It is used to identify and quantify proteins involved in a particular biological condition, and can also be applied to determine post-translational modifications, as well as cellular origin and place of action (Yates et al., 2009). The first proteomic studies were performed using gels to separate proteins. In the most commonly applied technique, two-dimensional electrophoresis (2-DE), proteins are separated according to their isoelectric points and then separated by their molecular weights. Thus, maps are created for each sample, where each spot corresponds to a protein or a small protein group. Using these maps, different conditions can be studied and compared through differential protein expression (up or downregulated; Zhu et al., 2003). To increase sensitivity and reproducibility, 2DE assays using fluorescence have been developed (differential electrophoresis; DIGE). This technique is robust because it allows the simultaneous analysis of more than one condition (multiplex), excluding the necessity of experimental replicates, and because fluorophores can detect protein concentrations more precisely than traditional methods (Coomassie blue and silver nitrate; Vranakisa et al., 2013). Proteomic assays are also performed to characterize and quantify post-translational modifications (PTM), which are essential for a range of protein functions, including infectious processes (Olsen and Mann, 2013). PTMs are detected by enrichment techniques using affinity antibodies, ionic interaction, or by specific enzymes (Zhao and Jensen, 2009).

Indeed, proteomic approaches have not been adopted in abundance in the study of the antibacterial MOAs of plant-derived compounds. A few examples are summarized in Table 1. In a recent study, the effects of tea polyphenols (TP; a mix of polyphenols extracted from green tea with a broad spectrum of bacterial activity) on protein expression in *P. aeruginosa* and *Serratia marcescens* were investigated by 2-DE and MALDI-TOF/TOF analyses. The results confirmed that the metabolic disorder caused by treatment with TP was associated with an increase in the amount of membrane proteins, such as chaperones and key enzymes involved in the control of membrane component biosynthesis pathways. These data supported a possible MOA for TP: increased membrane permeability leading to release of cellular components (Yi et al., 2010, 2014).

Plumbagin (naphthoquinone) is a yellow natural compound extracted from the *Plumbago zeylanica* L. root, which is widely used in traditional medicine in India and China. The antibacterial effect of plumbagin on *Bacillus subtilis* was evaluated by two complementary proteomic techniques, 2-DE and iTRAQ, which identified differential expression for 230 proteins, mainly involved in the citric acid cycle and heme biosynthesis. These results indicated that plumbagin blocks energy generation and also suppresses fatty acid biosynthesis. Repression of proteins directly linked to cell division (FTSA and SpoVG) was also observed (Reddy et al., 2015). Another example is the elucidation of the MOA for the alkaloid roemerine from *Papaver rhoeas* against *E. coli*, which was related to the inhibition of membrane permeability and sugar transporter proteins (Gokgoz and Akbulut, 2015).

The MOA of rhodomyrtone (isolated from *Rhodomyrtus tomentosa*) against methicillin-resistant *S. aureus* was also studied by 2-DE analysis, which revealed that this compound affects the expression of major classes of proteins involved in cell wall biosynthesis, cell division, protein degradation, stress response, virulence factors, energy production, and macromolecules biosynthesis (Sianglum et al., 2011). Another similar work showed that rhodomyrtone affected enzymes associated with major metabolic pathways of *Streptococcus pyogenes*, apart from its inhibitory actions on virulence genes, glyceraldehyde-3-phosphate dehydrogenase, cAMP, and streptococcal pyrogenic exotoxin C (Limsuwan et al., 2011). These works provided important insights into the antibacterial activity of this new natural compound, a promising candidate for a therapeutic agent against bacterial infections.

## Metabolomics analyses

The last “omics” technology to be presented in this review is metabolomics, which is defined as the study of the global profile of metabolites present in a biological system under certain conditions and time (Nambiar et al., 2010). “Metabolites” is a general term for a range of end products of cellular processes that belong to different classes, such as organic acids, amino acids, fatty acids, sugars, sugar alcohols, steroids, nucleic acid bases, etc. (Goodacre et al., 2004). Metabolomics analysis investigates biochemical disturbances caused by disease, drugs, toxins, and other factors such as genetic modification, etc. (Monteiro et al., 2013). The development of analytical and data mining methods is responsible for the quick evolution in the metabolomics field, providing more insights into cellular organization (Dunn et al., 2011). The methods followed to generate metabolic signatures are usually nuclear magnetic resonance (NMR), gas chromatography (GC), liquid chromatography (LC) coupled to mass spectrometry (LC/MS), capillary electrophoresis coupled to mass spectrometry (CE-DM), ultra performance liquid chromatography coupled to mass spectrometry (UPLC/MS), high-performance liquid chromatography-electrospray ionization coupled to mass spectrometry (HPLC-ESI/MS), high-performance liquid chromatography-diode array detection-electrospray ionization tandem mass spectrometry (HPLC/DAD/ESI-MS; Zhang et al., 2012). Multivariate analysis of statistical data needs to be performed owing the complexity of interpreting the data resulting from an analysis based on the metabolome. Principal component analysis (PCA) is the most prevalent method followed to reduce the dimensionality of the data, although other data mining techniques are routinely applied, such as clustering algorithms. By PCA, the data obtained are structured in a two/three-dimensional scores plot where the clustering of samples is shown in either similar or different groupings (Cox et al., 2014).

Metabolomics approaches have been widely adopted to investigate the responses of microorganisms to various environmental stressors such as heavy metals, temperature, and organic compounds (Lankadurai et al., 2013), and they are suitable tools for the study of metabolism disorders in microorganisms treated with antibiotics (Aliferis and Jabaji, 2011). Conversely, the application of metabonomic approaches for the discovery of plant-derived compound mechanisms of action is still largely unexplored, with *S. aureus* the most studied for this purpose (Table 1). In general, the metabolic profile induced by the tested compound is compared with those obtained using antibiotics with known action mechanisms. For example, the anti-*S. aureus* action of *Aquilegia oxysepala* extract (used in traditional Chinese medicine) and its main chemical components (genkwanin, apigenin, maguoflorine, and berberine) were investigated by metabonomics (HPLC/DAD/ESI-MS analysis combined with PCA) and compared with the profile induced by nine antibiotics with known modes of action. This study has shown that the *A. oxysepala* target should be similar to those of protein synthesis inhibitors (lincolmensin, erythromycin, chloromycetin, streptomycin, and acheomycin), and maguoflorine was the main component responsible for antibacterial activity (Yu et al., 2007a). In this study, berberine showed the same effects as rifampicin and norfloxacin, which are drugs that target nucleic acids (Yu et al., 2007a).

Similarly, the intracellular metabolic profiles of *S. aureus* treated with rhizome extract from *Tinospora capillipes* and its main constituents (columbin, palmatine, tinoside, and jatrorrhizine) were studied by HPLC-ESI/MS, showing the inhibition of RNA polymerase, gyrase, and topoisomerase IV as targets (similar to rifampicin and norfloxacin). Palmatite and jatrorrhizine were the most active compounds (Yu et al., 2007b). However, these metabolomic strategies, which compare the metabolic profiles of phytochemicals with known antibiotics, may be somewhat limited because they cannot be applied to define a new mechanism of action, but are viable and unexplored alternatives to assign known mechanisms to new bioactive compounds.

An interesting approach to be explored is the study of the exometabolome (metabolites that are secreted into the culture medium). Birkenstock et al. (2012), applying an exometabolome profiling approach, revealed that triphenylbismuthdichloride affects bacterial pyruvate catabolism, leading to the discovery of this compound as an efficient and non-competitive inhibitor of the bacterial pyruvate dehydrogenase complex. This study illustrates that metabolomics may represent a promising strategy to elucidate the MOA of bioactive compounds, and for combating multidrug-resistant bacteria.

## Conclusions

Taking into account all the possibilities provided by “omics” technologies, these approaches offer very exciting opportunities to elucidate the antimicrobial mechanisms of plant-derived compounds. These studies are essential for the use of the most promising compounds as therapeutic agents, and they could also lead to the identification of new targets, owing to the high degree of structure diversity found in phytochemicals. As shown in Table 1, there is a diversity of pathways involved in the MOA of plant-derived antimicrobial compounds. The design of new antibiotics with different action modes and structures will be also driven by these studies, and thus cross-resistance events will be avoided and development of resistance to these novel compounds will be delayed. It is also expected that, upon knowledge of the molecular mechanism, only compounds that have targets unrelated to host factors will be selected. Nevertheless, some barriers still need to be overcome prior to the widespread popularization for “omics” approaches, such as operating cost, complexity, and the wide dynamic range of the samples, which are being solved by the constant improvements made in the field and in bioinformatics technology. An integrated study using different “omics” technologies would be the ideal approach to elucidate the MOA of a natural compound since each technique has particularities with respect to its limitations and the potential of providing responses.

## Author contributions

BS, LD, MC, PP conceived of the study and participated in its design and coordination. MD and TD contributed in the Section “Proteomics assays.” JR and MG contributed in the Sections “Overview of some phenotypical methods used for elucidation of antibacterial action mechanisms” and “Gemomics advances in antibiotics research.” BS, LD, TN drafted the manuscript. All authors read and approved the final manuscript.

### Conflict of interest statement

The authors declare that the research was conducted in the absence of any commercial or financial relationships that could be construed as a potential conflict of interest.
